# Executive function underlies both perspective selection and calculation in Level-1 visual perspective taking

**DOI:** 10.3758/s13423-018-1496-8

**Published:** 2018-06-12

**Authors:** Adam W. Qureshi, Rebecca L. Monk

**Affiliations:** Department of Psychology, Edge Hill University, Ormskirk, UK

**Keywords:** Visual perspective-taking, Perspective calculation, Perspective selection, Theory of mind, Executive function, Dual-tasking

## Abstract

Previous research has suggested that the calculation of another’s perspective is cognitively efficient, whereas perspective selection (selection of a particular perspective, self or other) is associated with executive function, particularly inhibitory control. However, research has not previously tested how perspective calculation and selection may be associated with another key executive function, working memory. In the present study (*N* = 54 adult participants), we administered a Level-1 visual perspective task in a dual-task paradigm in which the secondary task required working memory. The results suggest that executive function is associated with both perspective calculation and perspective selection, contrary to previous evidence from similar dual-task studies that had used inhibitory control and attention-based secondary tasks. This contrast may suggest that working memory capacity facilitates perspective-taking. Furthermore, it may follow that the notion of simple perspective-taking is distinct from more the complex and cognitively demanding “theory of mind.” Research of this nature warrants further investigation.

Theory of mind (ToM) has been defined as the ability to understand that others have different beliefs, knowledge, and mental states from yours (Premack & Woodruff, 1978). Examinations suggest that ToM performance is correlated with executive function (EF) ability in both children (Apperly, 2012; Frick & Baumeler, 2017; Marcovitch et al., 2015; Müller, Liebermann-Finestone, Carpendale, Hammond, & Bibok, 2012) and adults (Fizke, Barthel, Peters, & Rakoczy, 2014; Phillips et al., 2011). A key aspect of this ToM capacity is the ability to correctly infer or determine another’s viewpoint (termed *perspective-taking*).

## Perspective taking in infants and adults

Research suggests that even though infants and young children possess limited executive resources (Onishi & Baillargeon, 2005; Sodian, Thoermer, & Metz, 2007), there is an early-developing ability to track what a target agent can or cannot see (Level-1 visual perspective taking [VPT]; Flavell, Everett, Croft, & Flavell, 1981; Moll & Tomasello, 2006). There may therefore be a distinction between perspective calculation and perspective selection (Leslie, German, & Polizzi, 2005; also see Leslie & Thaiss, 1992). The former is proposed to be a modular process that is fast, automatic and cognitively efficient. In contrast, selection requires effortful deployment of executive resources in order to select a given perspective (self or other).

Studies on adults (Fizke et al., 2014), including dual-task studies (Bull, Phillips, & Conway, 2008; McKinnon & Moscovitch, 2007), suggest that EF is associated with ToM. Converging evidence has also been found in older adults (Bradford, Brunsdon, & Ferguson, 2016, 2017; Phillips et al., 2011) and patient studies (Apperly, Samson, & Humphreys, 2005), all of which indicate that impaired executive abilities are associated with reduced performance on a range of ToM tasks (though see Bird, Castelli, Malik, Frith, & Husain, 2004).

Whereas some studies have suggested that EFs explain perspective-taking abilities (e.g., spatial cueing; Santiesteban, Kaur, Bird, & Catmur, 2017) in a “submentalizing” approach, others disagree (Michael et al., 2018) and propose an “implicit mentalizing” ability. As such, a distinction is postulated between calculating what someone knows/sees and selecting that information in order to make a decision (Qureshi, Apperly, & Samson, 2010). Accordingly, EF (specifically, inhibitory control; IC) is apparently needed for perspective selection but not perspective calculation (Qureshi et al., 2010). This supports the contention of Leslie et al. (2005) that perspective calculation and selection are separate processes, and that whereas the former is automatic, the latter requires more effortful (executive) resources. Indeed, a recent study by Todd, Cameron, and Simpson (2017) used a process-dissociation procedure (PDP) to show that in a Level-1 perspective-taking task, automatic processing of a target’s perspective was unaffected by time pressure, whereas controlled processing of one’s own responses was impaired. This again suggests that there is an automatic component of perspective calculation.

Working memory is another EF that has been suggested to be linked to perspective taking (e.g., Reed, 2002), and it has been examined in relation to more complex ToM tasks (e.g., story-based tasks and the Reading the Mind in the Eyes Test; Bull et al., 2008; McKinnon & Moscovitch, 2007). It has also been investigated in tasks that involve perspective taking (Cane, Ferguson, & Apperly, 2013; Meyer & Lieberman, 2016), with results suggesting that WM is involved. However, to our knowledge, its potential contribution to the components of perspective taking, namely perspective calculation and selection, has not been tested previously.

The present study uses the same Level-1 VPT task (Samson, Apperly, Braithwaite, Andrews, & Bodley Scott, 2010)^1^ used by Todd et al. (2017). This requires participants to judge what can be seen by themselves or another agent whose physical orientation means that he or she may not see the same set of objects as the participant.

### Predictions

The dot task requires participants to judge and answer questions regarding the number of dots currently in view to themselves (*self* perspective) or a displayed avatar (*other* perspective; see the Method section for more information). The avatar is positioned so that the content of their perspective can be the same as that of the participant (consistent) or different (inconsistent). Previous studies have shown that performance in the inconsistent conditions, for both *self* and *other* judgments, are impaired relative to the consistent condition. When judging the avatar’s perspective, this interference comes from the *self* perspective (termed *egocentric interference*), and is expected because of the salient nature of one’s own perspective. When judging the *self* perspective, this (altercentric) interference comes from the avatar perspective, suggesting that the avatar’s perspective is processed despite it being irrelevant.

It has been suggested that perspective selection is the process that is involved when dealing with such interference and it is purported to require effortful resources (such as IC, the ability to withhold a dominant response; Qureshi et al., 2010), whereas perspective calculation (calculating what someone knows and sees) is a more automatic processes. As such, taxing WM in the dual-task condition (by requiring participants to hold letters in mind until after they respond) should impact perspective selection and spare perspective calculation. A number of hypotheses result when using this task. They are as follows and are largely informed by Qureshi et al.:

#### Perspective selection

If working memory is needed for perspective selection, then performance in the dual-task condition on inconsistent trials should be further impaired relative to consistent trials, resulting in higher *egocentric* and *altercentric* interference effects, as compared to these effects when WM is not taxed.

#### Perspective calculation

If working memory is required for the calculation of the avatar’s perspective, then this will be disrupted in the dual-task condition. Since the irrelevant calculation of the avatar’s perspective is suggested to be the reason for the interference effect shown in inconsistent *self* judgments, this disruption should result in the reduction of this interference effect, meaning that the *altercentric* interference effects will be reduced.

In contrast, for egocentric interference (in which calculation of the *self* perspective interferes with *other* judgments) it is predicted that interference would remain the same in the dual-task condition in the standard dot task, or else be increased. Additionally, if working memory is involved in the calculation of the avatar perspective, then processing costs of all *other* judgments would be expected to increase in the dual-task condition. These predictions are summarized in Table 1.

**Table 1:** Summary of predictions for the effects of concurrent performance of an executive function task on *self* perspective and *other* perspective if executive function is necessary for perspective calculation or perspective selection

	Role of executive function	
	Calculation	Selection
*Self* judgments	Decreased altercentric interference	Increased altercentric interference
*Other* judgments	Increased processing cost for all *other* judgments	Increased egocentric interference

The hypotheses for the PDP analyses are that the automatic processes will not be affected by working memory, whereas the controlled processes should be. Additionally, the data from Qureshi et al. (2010) was analyzed using a PDP approach, with the same hypotheses, in order to illuminate any differences between working memory and inhibitory control in their effect on automatic and controlled processes in the L1 VPT task.

## Method

### Participants

Fifty-four students (47 female, seven male; all right-handed; mean age = 19.03, range = 18–22) participated in the study for course credits. Informed consent was obtained from all participants. On the basis of effect sizes obtained in Qureshi et al. (2010), with observed power of .95 and alpha set at .05, a minimum of nine participants were required.^2^

### Apparatus

All tasks were presented on a 19-in. Dell 1079FP monitor and a standard keyboard with a desktop PC in a testing laboratory, using E-Prime 2.0 (PST Inc.).

### Design

#### Visual perspective task

The VPT task used the stimuli and procedure of Samson et al. (2010, Exp. 1). The main stimuli consisted of a picture of a laterally viewed room with the left, back and right walls visible and with red dots displayed on one or both the left and right walls (Table 2). A centrally positioned human avatar of the same gender as the participant faced either the left or right wall. In 50% of trials, the avatar’s position meant that s/he saw the same dots as the participant (consistent condition), whilst in the remaining trials their position meant they did not (inconsistent condition). Participants were cued as to which perspective to take (“YOU” vs. “S/HE”) and as to what number of dots to verify (between 0 and 3). These cues were simultaneously presented for 750 ms. The room was then shown until participants responded (or a maximum time of 2,000 ms). Participants responded by pressing “Q” on the keyboard for “yes” and “P” for “no.” The main task consisted of four blocks of 52 trials.

**Table 2:**
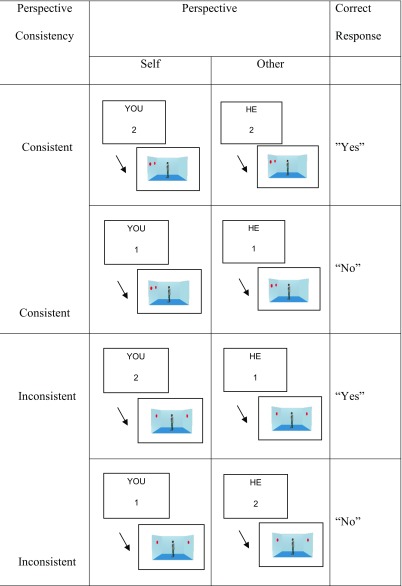
Example event sequences for the different conditions of the visual perspective-taking task

#### Executive task

A forward letter span task was used to tap working memory (Zirk-Sadowski, Szucs, & Holmes, 2013). The digits were presented using headphones, and participants responded using the keyboard. The task to establish each participant’s maximum letter span was completed alone, prior to the main experiment. Here, participants initially completed three trials of three letters. If they successfully recalled two or more of these, the letter span was increased to by one letter to a maximum of nine. If participants only recalled one of the three trials, the letter span was reduced by one (to minimum of two). A total of seven runs of three trials were conducted, with the maximum span deemed to be the highest span for which they had successfully recalled two or more of the three trials. In the dual-task condition, the letters were presented prior to each VP trial. Participants were required to hold the letters in mind until after they responded to the L1 VPT trial, at which point they responded.

### Procedure

Participants initially completed the letter span task alone. The starting span length was three, and this was increased according to participant accuracy until their maximum letter span was established (to maximum of nine). This value, minus one, was used for the dual-task condition in both the practice and experimental trials, in order to have a consistent working memory demand, whilst avoiding potential floor effects. Participants then completed ten practice trials of the VP task in the alone task condition, followed by ten practice trials in the dual-task condition. The orders of blocks and task conditions were counterbalanced.

## Results

### Visual perspective task

Accuracy rates were calculated for each participant by condition. As per Samson et al. (2010), only matching trial data was analyzed (in which the correct response was “yes”). Prior data screening removed any response times more than 2.5 *SD* from the mean, by condition. Omissions resulting from participants timing out were also removed.

A repeated measures analysis of variance (ANOVA) was conducted with the following independent variables (and levels): consistency (consistent, inconsistent), perspective (self, other), and task condition (alone, dual). The analyses showed a main effect of task condition [*F*(1, 53) = 38.40, *p* < .01, *η*_p_^2^ = .42], with significantly higher error rates in the dual-task condition. There was also a main effect of consistency [*F*(1, 53) = 51.04, *p* < .01, *η*_p_^2^ = .49], with more errors made in the inconsistent condition.

**Fig. 1:**
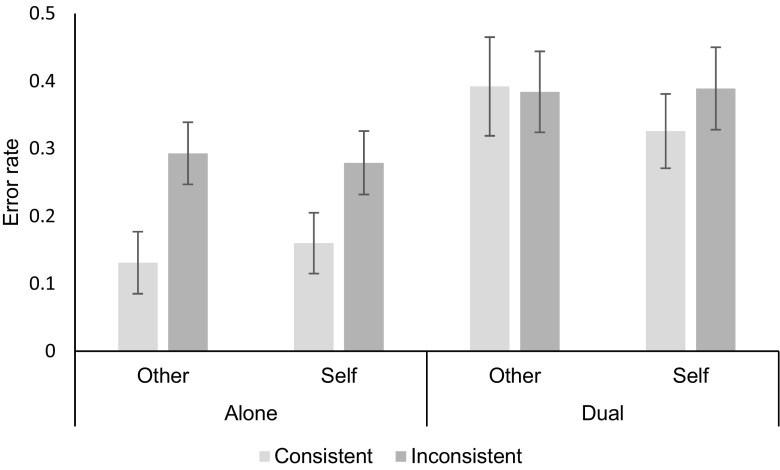
Error rates by task condition, consistency, and perspective (bars = confidence intervals)

We observed an interaction between task condition and consistency [*F*(1, 53) = 23.47, *p* < .01, *η*_p_^2^ = .31], as well as a three-way interaction between task condition, consistency, and perspective [*F*(1, 53) = 6.10, *p* < .05, *η*_p_^2^ = .10; see Fig. 1].

Simple main effects showed that error rates for the dual task were significantly higher for all conditions (*p*s < .01). Error rates for inconsistent conditions were significantly higher than for consistent conditions (*p*s < .01), except for *other*-perspective trials in the dual-task condition, in which there was no consistency effect (*p* = .79). Error rates for *self* and *other* perspectives were not significantly different for all conditions (*p*s > .14) except for consistent trials in the dual task condition, in which the error rate for *other*-perspective trials was significantly higher (than *self*-perspective trials; *p* < .01).

### PDP analyses

Calculation of controlled processes (C) and automatic processes (A) followed the procedure outlined by Todd et al. (2017). When looking at consistent trials, automatically calculating the avatar’s perspective (A) and explicitly responding with one’s own perspective (C) results in the probability of responding correctly being the sum of the probability of C and the probability of A when C fails: *P* (correct ǀ consistent trials = C + A (1 – C). When trials are inconsistent, the controlled and automatic processes result in different responses. Therefore the probability of an incorrect response is the probability of the automatic processing operating when the controlled process fails: *P* (incorrect ǀ inconsistent) = A (1 – C). Using these equations, estimates of C and A can be calculated:

$$ {\displaystyle \begin{array}{l}C=P\left(\mathrm{correct}|\mathrm{consistent}\kern0.5em \mathrm{trials}\right)\hbox{--} P\left(\mathrm{incorrect}|\mathrm{inconsistent}\ \mathrm{trials}\right)\\ {}A=P\left(\mathrm{incorrect}|\mathrm{inconsistent}\ \mathrm{trials}\right)/\left(1\hbox{--} \mathrm{C}\right)\end{array}} $$

#### Analyses of data from Qureshi et al. (2010)

These analyses showed that C was weaker in the inhibitory control dual-task condition (*M* = .66, *SD* = .15) than for the alone condition (*M* = .83, *SD* = .10) [*t*(29) = 7.17, *p* < .01, *d* = 1.40]. A was not different between the dual-task and alone conditions (dual: *M* = .65, *SD* = .19, alone: *M* = .56, *SD* = .18) [*t*(29) = – 2.04, *p* > .05, *d* = – 0.37]. These results suggest that inhibitory control may not be associated with calculation of the avatar perspective (A),^3^ but is involved in responding with one’s own perspective (C).

#### Analysis of data from current study

For the present study, C was weaker in the WM dual-task condition (*M* = .28, *SD* = .39) than in the alone condition (*M* = .55, *SD* = .29) [*t*(53) = 5.04, *p* < .01, *d* = .70]. The same pattern was shown for A (dual-task: *M* = .54, *SD* = .14; alone: *M* = .64, *SD* = .15) [*t*(53) = 3.89, *p* < .01, *d* = 0.53], but to a lesser degree. This suggests that WM is associated with both the controlled process of responding with one’s own perspective, but also with the seemingly automatic process of (calculating) the avatar perspective.

### Executive task

Participants’ maximum letter span ranged from 3 to 7. The mean accuracy for the letter span task in the dual-task condition was .74 (*SD* = .21). Performance was analyzed using a repeated measures ANOVA, with conditions of consistency and perspective. The analyses showed only a main effect of perspective [*F*(1, 53) = 7.49, *p* < .01, *η*_p_^2^ = .12], with accuracy being lower for *other*-perspective trials (*M* = .71, *SD* = .20) than for *self*-perspective trials (*M* = .77, *SD* = .17).

Performance on the letter span, when completed alone, was compared to letter span performance in the VP task dual condition using paired sample *t* tests (compared with their letter span accuracy^4^ in the alone condition). These showed that performance in the alone condition was better in all conditions, and significantly so for all except consistent *self* trials: consistent *other* trials [*t*(53) = 3.29, *p* < .01, *d* = – 0.65], consistent *self* trials [*t*(53) = – 1.82, *p* = .08, *d* = – 0.35], inconsistent *other* trials [*t*(53) = – 3.41, *p* < .01, *d* = – 0.68], and inconsistent *self* trials [*t*(53) = – 2.36, *p* < .05, *d* = – 0.47].

### Strategic trade-off

The possibility of any strategic trade-off between performances in the VP and digit span tasks was checked using *μ* scores (where *μ* = {1 – [(pm + pt)/2]} × 100, pm = proportional loss on the primary task and pt = proportional loss on the secondary task). A score of 100 indicates no deterioration in performance under dual-task conditions; the lower the score, the greater the dual-task performance detriment. When the primary and secondary tasks share the same cognitive processes, *μ* scores should be lower than when the tasks do not share the same resource.

Proportional loss of performance on the VP task was calculated for each condition as [(dual – alone) accuracy rate]. Proportional loss of performance on the digit span task was calculated as the difference between their dual task recall and their alone performance (higher scores = higher proportional loss of performance). The *μ* score for each condition is shown in Table 3.

**Table 3:** Mean *μ* score for each task condition

	Consistent, Other [*M* (*SD*)]	Consistent, Self [*M* (*SD*)]	Inconsistent, Other [*M* (*SD*)]	Inconsistent, Self [*M* (*SD*)]
Mean *μ* score	91.91 (22.43)	98.18 (14.77)	100.41 (20.04)	97.77 (16.85)

The scores indicate that the tasks share the same cognitive processes and that there is no strategic trade-off, though WM seems to be more needed for consistent *other* trials.

To test the contribution of working memory capacity to performance in the VP task, participants’ maximum letter span was entered as a covariate in a replication of the main analyses. No effects were significant (all *p*s > .10), suggesting that working memory capacity could account for differences between task conditions as well as for the interaction of task condition and perspective.

Results from the PDP analyses on data from Qureshi et al. (2010) suggest that inhibitory control is involved in the controlled process of responding with one’s own perspective, but not in the automatic calculation of the avatar perspective. The same analyses on the present data suggest that working memory is involved in both the controlled and automatic processes in the L1 VPT task. The response time analyses (see the Appx.) show an increase in response time in the dual-task condition for both consistent and inconsistent *self* and avatar judgments, but disproportionately so for consistent judgments. Again, this may suggest a role of working memory in perspective calculation.

## Discussion

Performance of an executive task in conjunction with a VPT task increased overall processing costs, but disproportionately increased these when making *other* (as compared to *self*) judgments. This suggests a heightening of processing costs that is unlikely to be caused by general task demands, motor planning or motor execution processes. Instead, these results may posit a role for working memory in the calculation of the avatar perspective, but not in perspective selection.

PDP analyses on the present task suggest that *both* controlled and automatic processes are impaired in the dual-task condition, which places the greatest strain on working memory. This research is the first of its kind to examine working memory’s potential contribution to perspective taking and selection. Nevertheless, the present findings are somewhat contrasting with previous research that has suggested that other facets of EF (e.g., inhibitory control or attention) may be important for perspective selection, whereas calculation is automatic, independent of EF (Michael et al., 2018; Qureshi et al., 2010), and unaffected by time demands (Todd et al., 2017). Indeed, the present findings seem to indicate that although particularly important for perspective selection, working memory may be imperative for both calculation and selection.

Specifically, in the present study, standard analyses indicate that performance is most detrimentally affected on consistent *other*-perspective trials (as the *μ* scores corroborate), which may suggest that this task is most reliant upon working memory. It may therefore be postulated that working memory contributes to the calculation of avatar perspective, though it is unclear if it is a prerequisite of calculation or if it merely facilitates this process. Bradford, Jentzsch, and Gomez (2015) argued that the *self* perspective is taken automatically, and that this forms the basis of taking an *other* perspective—which requires cognitive effort and is only done when explicitly required. Alongside the present findings, we therefore propose that a greater working memory capacity may allow for a better ability to hold the basis of the (automatically taken) *self* perspective in mind, in order to then process the *other* perspective (cf. mental rotation, Hyun & Luck, 2007; holding competing perspectives in mind, Olson, 1993). Therefore, although working memory may be more important in processing the avatar’s perspective, there remains some apparent role for memory in the perspective calculation process.

### Conclusion

In summary, *other* judgments were selectively disrupted in the dual condition. In conjunction with prior findings for inhibitory control and PDP analyses, the present research suggests a role for executive resources in perspective calculation and perspective selection. More specifically, it suggests that working memory may drive both selection and calculation, with a particularly important role in perspective calculation, whereas inhibitory control seems to be significant in perspective selection. These results complement the literature on the cognitive processes underlying ToM in both adults (Fizke et al., 2014; Phillips et al., 2011) and children (Carlson & Moses, 2001; Carlson, Moses, & Breton, 2002; Powell & Carey, 2017). However, that infants show perspective-taking abilities (Baillaregeon, Scott, & He, 2010; Sodian et al., 2007; Onishi & Baillargeon, 2005) may suggest that working memory may *facilitate* perspective taking, but is not the sole driver of this capacity (cf. Roncadin, Pascual-Leone, Rich, & Dennis, 2007). In other words, because the ability to calculate perspectives is present in infants with little or no working memory capacity, we suggest that perspective may be a base capacity but one that becomes more efficient when working memory develops and suffers when executive resources are stretched.

If working memory does only facilitate cognitively efficient perspective calculation, results provide partial support for the distinction between relatively cognitively efficient perspective calculation and a more cognitively demanding perspective selection (cf. Leslie et al., 2005; Leslie & Thaiss, 1992). More generally, these results may also highlight the wider distinction between a more cognitively efficient simple perspective-taking ability and the more cognitively demanding ToM ability (Apperly & Butterfill, 2009). However, further research is needed to establish whether these are separate abilities or whether the simple perspective-taking ability forms the core of ToM, that then grows as executive capacity increases throughout development.

### Limitations and further work

The interpretation of these results is that WM is necessary for perspective calculation and perspective selection. However, the authors acknowledge that more research is necessary in this area to firm up support for this assertion. Specifically, we note that the nature of the incidental task demands should be explored further. In the present study, participants needed to count the discs and compare them to the target number. Although the simultaneous presentation of both the perspective and content cues was intended to lower WM load, we cannot rule out that this task may have exerted some pressure on WM. As such, it may be that the counting task, as opposed to the perspective taking itself, that required WM. Future research should therefore utilize a perspective-taking task that has lower or no WM task demands (e.g., the task of Kovács, Téglás, & Endress, 2010). This would allow further elucidation of the role of WM in perspective taking without any possible contamination from incidental task demands. It is also possible that motor interference between the primary and secondary task responses may have occurred, though as the response for the L1 VPT was less complex than for the letter span, any interference should have been minimal (Ulrich et al., 2006). More implicit measures (e.g., eye-tracking) could be implemented in the future. The present study only utilized letter span measures of working memory. Spatial measures such as the Corsi Block Test may shed more light on the relationship between working memory and perspective taking. Finally, whether the proposed relationship holds for more complex perspective taking (e.g., Level 2) should also be explored.

## Figures and Tables

**Fig. 2:**
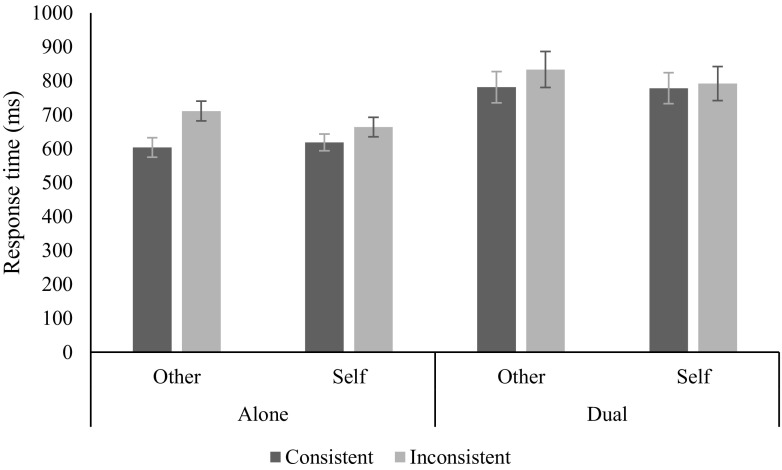
Response times (in milliseconds) by task condition, consistency, and perspective (bars = confidence intervals)

## Appendix: Response time analyses

The same initial data screening was conducted as for the error rate analyses. A repeated measures ANOVA was conducted with the following independent variables (and levels): consistency (consistent, inconsistent), perspective (self, other), and task condition (alone, dual).

We found a main effect of task condition [*F*(1, 52) = 60.84, *p* < .01, *η*_p_^2^ = .54], with longer response times in the dual-task condition. There was also a main effect of consistency [*F*(1, 52) = 62.96, *p* < .01, *η*_p_^2^ = .55], with longer response times for inconsistent trials. A main effect of perspective emerged [*F*(1, 52) = 7.88, *p* < .01, *η*_p_^2^ = .13], with response times being marginally higher for *self* judgments. There were also interactions between task condition and consistency [*F*(1, 52) = 9.31, *p* < .01, *η*_p_^2^ = .15] and between consistency and perspective [*F*(1, 52) = 21.00, *p* < .01, *η*_p_^2^ = .29]. These were analyzed further using simple main effects (see Fig. 2).

For the interaction between task condition and consistency, response times in the dual condition were longer for both consistent and inconsistent trials (*p*s < .01). Inconsistent trials had longer response times than did consistent trials in both the alone and dual-task conditions (*p*s < .01), but this difference was reduced in the dual condition (dual mean difference = 32.76 ms, alone mean difference = 76.10 ms).

For the interaction between consistency and perspective, inconsistent trials had longer response times for both *other* and *self* judgments (*p*s < .01), but this difference was greater for *other* judgments. Although *other* judgments had longer response times on inconsistent trials than did *self* judgments (*p* < .01), there was no difference between judgments on consistent trials (*p* = .47).

## Footnotes

1 based on the task used by Sodian et al. (2007), using 14-month-old children in conjunction with a secondary task that required inhibitory control in a dual-task design

2 Due to the uncertainty of the actual effect sizes or observed power required for the present study, we decided to overrecruit. Post-hoc sensitivity analyses suggest that the current sample size was sufficient to detect a medium effect (*f* ~ 0.2) with an observed power of .8 (alpha = .05).

3 A power analysis suggests that the probability of detecting a significant effect is .5, so it is possible that inhibitory control may be associated with calculation of the avatar perspective.

4 for their maximum span minus 1 (the value used in the dual-task condition)
